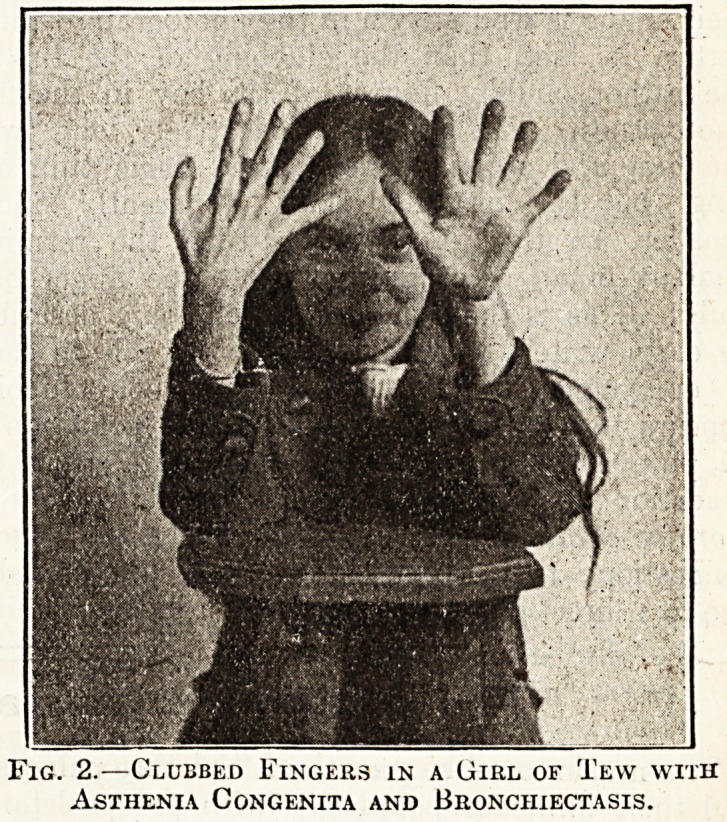# An Aid to Prognosis for the School Doctor

**Published:** 1914-04-25

**Authors:** 


### CLUBBED FINGERS IN CHILDREN.

### An Aid to Prognosis for the School Doctor.

In two conditions the state of the finger tips is of some importance in formulating a prognosis as to the child's future at school. It must be remembered that the school doctor is in a slightly more invidious position than the family practitioner; he must give an opinion not so much as regards the child's chances of life, but as regards its chances of such health as will enable it to benefit by the teaching and training it receives in an elementary school. In one sense his responsibilities are more limited; in another they are wider; and it is only by paying attention to minor points that he can, nearly always, take a safe measure between two extremes and a given prognosis which is justifiable and safe. One of these smaller points, which is usually of definite diagnostic importance, is the condition known as clubbed fingers. In a minor degree it is by no means infrequently met with in school children who suffer or have suffered from such diverse illnesses as rheumatic fever, appendicitis, latent exophthalmic goitre, and mumps,

Fig. 1.—Clubbed Fingers in a Boy of Eight with Congenital Heart Disease.

but in such minor degree it rarely attracts attention, even at the routine inspection. In these lesser stages of the deformity, the clubbing is not sharply defined, and there is no striking differentiation between the second and terminal phalanges, nor, usually, is there any prominent physical defect that needs prognosis. But sometimes one meets with the deformity in an advanced degree of development. Such advanced cases are chiefly children afflicted with cardiac or pulmonary defects, the two most common defects being congenital heart disease and bronchiectasis.

The annexed photographs show an almost typical example of the finger tips in these two conditions. The first photograph shows the hands of a little boy of eight, who is a sufferer from congenital heart disease. His face is puffed, cyanotic, with the superficial venules well marked; he is out of breath on the slightest exertion; he is, as most children with heart defects are, under-developed, with thin bones and weak thorax, although his muscular development is not very much below the average. It must be remembered that in young children a cardiac defect revenges itself, if one may be allowed to use the analogy, not upon the vascular system in particular, but upon the whole development of the child. This is perhaps Nature's compensation for a weak heart in early life; she keeps the body small to obviate the necessity for greater effort on the part of the heart. Even when the degree of cardiac incompetency is relatively great in children, one rarely sees such gross evidences of overstrain and heart fatigue or actual heart failure as one would expect to find, from one's experience of adult cases, in these children. On the other hand, one sees marked and striking disturbances of development, and from

Fig. 2.—Clubbed Fingers in a Girl of Tew with Asthenia Congenita and Bronchiectasis.

such disturbance in development rather than from actual evidence of failure of compensation, one has to judge the individual's chances in after-life. As a general rule it may be taken that a child with well-marked congenital heart disease has a poor chance, both as regards life and as regards health. Such a child is certainly not fit for an ordinary elementary school, but must be relegated to a special school for physically defectives, where special care can be taken to obviate any undue strain upon him or her. Where, in such a case, marked clubbing of the fingers exists, the prognosis is far graver, and it is doubtful whether such a child ought to be allowed to attend school at all. The obvious treatment in such cases is to send the child to a home of recovery. Unfortunately we do not possess many such homes; there are, we believe, at present only two such homes in the kingdom, and there accommodation is necessarily very limited. In the case illustrated above the boy attends the infants' department; his intellectual development is that of a child of six; and he is a constant source of anxiety to the class teacher. Yet he is no better, but rather worse, at home, and probably his attendance at school does little harm, since he is in a measure supervised, whereas if he were excluded he would only be permitted to run about the street and be exposed to greater strain with the danger of sudden breakdown.

The pulmonary cases are on an entirely different footing. Fig. 2 shows such a case. It is that of a girl of ten, who is what Professor Stiller would call a congenital asthenic child with a marked *habitus asthenicus*, and with a strong family history of enteroptosis and tuberculosis. She has suffered since the age of five from attacks of chronic bronchitis; both lungs are bronchiectatic; she has a constant cough, but there is no definite evidence of tuberculosis, although, on the history and the clinical course of her last attack, her family practitioner diagnosed her as suffering from phthisis. Her fingers are definitely clubbed, and their shape is well shown in the photograph—much better, in fact, than the clubbing of the tips in the rather chubby fingers of the boy in the first photograph. The fingers are relatively long, with the first and second bones of the finger thin and long, but the terminal bone apparently much broader, so that it appears to be the terminal phalanx of an adult grafted on to the finger of a child. This is the condition of clubbing described by early writers in medicine as noticeable in cases of empyema. It is stated that such clubbing appears two or three weeks after the formation of an empyema and gradually disappears after the avacation of the pus. The clubbing in cases of *morbus cordis* is due, most probably, to venous congestion; that in pulmonary cases is probably due to a direct stimulation of growth in the terminal digits. The former is therefore a sign of active trouble; the latter may or may not be due to still active disease, and the prognosis in the pulmonary condition must therefore depend very largely on the clinical evidence elicited by auscultation and percussion. Again, as a general rule, one may state that such children, who show clubbing and who are chronic bronchiectatics, are scarcely fit pupils for an ordinary school. They, too, demand a home of recovery, and under proper conditions of hygiene they recover remarkably well, except where the pulmonary condition is complicated, as it was in the case illustrated above, by *asthenia congenita universalis*.

To the teachers, and, indeed, generally to the parents as well, these children are a source of anxiety, as they are regarded as "consumptive." So indeed they are, in a broad sense of the term, but it is often impossible to notify them, on the evidence, as cases of phthisis. The bronchiectasis may be well marked, and the amount of expectoration emitted by the child may be copious; for these reasons exclusion is called for in the interests of the school, but it is scarcely in the interests of the child to diagnose phthisis in such cases and to prescribe accordingly. Open-air treatment at a home of recovery often works wonders for these children; they put on weight, their appetite increases, their general health improves markedly, and the bronchitis diminishes. Yet these children remain, for the most part, invalids their life long, and the main question that confronts the school doctor is that of employment for them after they have left school. Such cases should be carefully noted and followed up. We know relatively little about the after-life history of these children with clubbed fingers, but experience shows that under proper treatment they do very well on the whole, and that the prognosis, as regards life, is by no means bad.

## Figures and Tables

**Fig. 1. f1:**
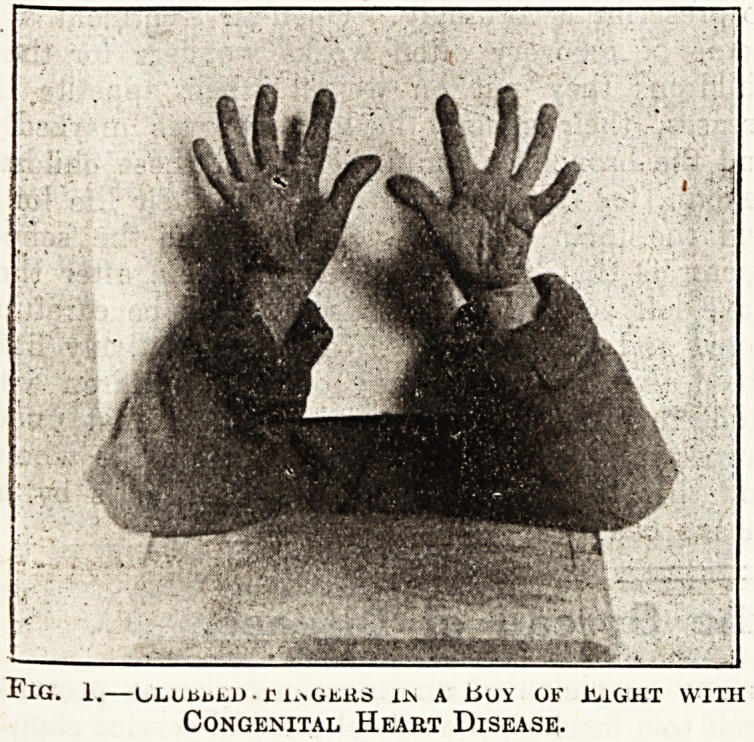


**Fig. 2. f2:**